# Emulsions, Preparation of

**Published:** 1874-12

**Authors:** 


*October:*

Emulsions.—Dr. Ernest C. Saunders, of Detroit, in the *Peninsular Journal of Medicine,* says: A few hints on the above subject may not be unacceptable to some unfortunate who has not had the privilege of being taught this important branch of our art thoroughly. The first thing to do is to see that the mucilage is fresh and sweet; for although good emulsions can be made with sour mucilage, they require more labor, and spoil more quickly. A broad, flat pestle will be found to serve better than a narrow, round-faced one. Be sure that the mortar is clean and free from grease. Then put in the mortar, first, a small quantity of the mucilage, and rub it round the mortar, so as to prevent any of the oil from adhering to the side. Add a little of the oil—about half the quantity of oil that you have used mucilage—and rub from the center; the emulsion will begin to form immediatel.y When the first quantities are thoroughly emulsified, add first more mucilage, and again half the quantity of oil, and make into a perfect emulsion. Continue in this way until all the oil is emulsified, adding water between each addition of oil after the right quantity of mucilage has been added. Great care must be taken to keep the mucilage and water in excess of the oil used, or a thick mass will be formed, which it will be impossible to mix with water. The object in making an emulsion is to have the particles of oil separated by water; but if the oil is in excess, the opposite is liable to take place—the particles of water are separated by the oil, and it is then impossible to form a good mixture, and the shortest way to do will be to throw out the mass and start again with fresh materials. Some people are in the habit of mixing the oil with powdered gum arabic, but it is impossible in that way to obtain a permanent emulsion, or even one in which the oil is sufficiently divided as to render the globules of oil invisible to the naked eye. A still worse mode is to put the oil and mucilage together in a bottle and shake them. A perfect emulsion should be as white as milk, if made with olive oil, or any light yellow or colorless oil; should mix readily with water in any proportion, without showing any signs of separating on standing, and should leave the mortar, or any vessel it may be put in, in such a condition that simple rinsing with cold water will clean the vessel, without leaving any traces of oil having been in it. The amount of mucilage to be used to a given quantity of oil varies. Half an ounce of mucilage is sufficient for two ounces of castor oil, or balsam of copaiba. Oil of turpentine, and the other light volatile oils, require rather more mucilage and longer trituration. If any syrup or sugar be ordered, it should be added after diluting the emulsion with all the water allowable. The same precaution should be taken with tinctures, or any alcoholic preparations. The fact that mucilage of acacia is precipitated by alcohol should always be borne in mind. It is difficult to give an unvarying rule as to the amount of tinctures admissible in one emulsion, as the amount of gum and oil varies so much; but, as a rule, it is unsafe to put more than one ounce of a tincture made with diluted alcohol in a four ounce emulsion, and tinctures made with stronger alcohol in proportion. Emulsions, if well made, are very handsome mixtures and very permanent. The writer has some emulsions of castor oil made four weeks ago, and of balsam copaiba made three weeks ago, both of which are as fresh and nice as the day they were made, and neither of which show the least sign of separation.

